# Cleidocranial Dysplasia With Multiple Impacted Teeth and Dentigerous Cysts: A Case Report of a Rare Entity

**DOI:** 10.7759/cureus.89098

**Published:** 2025-07-30

**Authors:** Manhar Shinh

**Affiliations:** 1 Department of Dentistry, Adesh Institute of Dental Sciences and Research, Bathinda, IND

**Keywords:** cleidocranial dysplasia, dentigerous cyst, mutation, runx2, supernumerary teeth

## Abstract

This report describes an unusual case of a 27-year-old male presenting with cleidocranial dysplasia (CCD), a rare genetic disorder affecting bone and dental development. The patient exhibited classic features, including short stature, drooping shoulders with hypermobility, broad forehead, maxillary deficiency, and mandibular prognathism. Intraoral examination revealed multiple missing teeth, retained deciduous teeth, and gingival swelling in the maxillary anterior region. Radiographic evaluation, including orthopantomogram and cone beam CT (CBCT), revealed 45 impacted permanent and supernumerary teeth, a rare finding, along with multiple dentigerous cysts, notably a large cyst in the right maxilla causing cortical plate breaches and nasal cavity displacement. Additional skeletal abnormalities included hypoplastic clavicles, open cranial sutures, and underdeveloped sinuses. Genetic testing confirmed a Runt-related transcription factor 2 (*RUNX2* gene) mutation, thus confirming the diagnosis.

The extensive number of impacted teeth and cystic lesions sets this case apart from the typical presentations, which usually involve fewer supernumerary teeth and rare cystic changes. The complexity of dental and skeletal anomalies necessitates a multidisciplinary approach involving oral surgery, orthodontics, and prosthodontics to restore function and aesthetics. Early diagnosis through clinical, radiographic, and genetic assessments is crucial to prevent complications, such as cyst expansion or bone resorption. This case emphasizes the importance of comprehensive evaluation and tailored treatment planning in managing the severe manifestations of CCD, highlighting its diverse clinical spectrum and the need for individualized care.

## Introduction

Cleidocranial dysplasia (CCD) is a rare autosomal dominant skeletal dysplasia characterized by a spectrum of skeletal and dental anomalies [1]. This congenital disorder, also known as Marie-Sainton disease or cleidocranial dysostosis, arises from mutations in the Runt-related transcription factor 2 (*RUNX2* gene; previously known as core binding factor subunit alpha or CBFA1) located on chromosome 6p21 [2]. RUNX2 is pivotal for osteoblast differentiation and bone formation, influencing both membranous and endochondral ossification processes. Mutations in this gene disrupt normal bone development, particularly affecting bones that ossify early, such as the clavicles, which are typically hypoplastic or absent in patients with CCD [2,3]. Nevertheless, 40% of CCD cases manifest without discernible genetic etiology. This condition predominantly affects skeletal structures, resulting from endochondral and intramembranous ossification processes, including the skull and clavicular bones. The diagnostic process relies on clinical and radiographic evaluations [2]. Its prevalence is estimated to be 0.5/100,000 live births, affecting both sexes equally, without any racial predilection [4].

The hallmark features of CCD include hypoplasia or aplasia of the clavicles, delayed closure of the cranial sutures and fontanelles, short stature, and various dental abnormalities [2]. These dental manifestations are particularly distinctive, encompassing retained deciduous teeth, delayed eruption of permanent teeth, and the presence of multiple supernumerary teeth, which often lead to impaction. In addition, dentigerous cysts may form around impacted teeth, further complicating the dental management [5]. Skeletal anomalies extend beyond the clavicles to include a widened pubic symphysis, underdeveloped paranasal sinuses, and anomalies in the hands and spine, such as clinodactyly and scoliosis, respectively. These features contribute to characteristic physical traits, such as hypermobile shoulders, brachycephaly, frontal bossing, hypertelorism, and mid-facial hypoplasia, which are often evident on clinical examination [5,6].

The pathophysiology of CCD is linked to defective osteoblast differentiation, which impairs bone remodeling and tooth eruption. The lack of cellular cementum and mechanical obstruction by supernumerary teeth are significant contributors to tooth eruption [7]. Radiographic evaluations, including orthopantomography (OPG) and cone-beam computed tomography (CBCT), are crucial for determining the extent of dental and skeletal abnormalities [8]. This report presents a rare case of CCD with 45 impacted permanent and supernumerary teeth and multiple dentigerous cysts, highlighting the complexity of its clinical presentation and the need for a multidisciplinary management approach. This study aimed to describe the clinical and radiographic features of a rare case of CCD, emphasizing the importance of early diagnosis and comprehensive treatment planning.

Written informed consent was obtained from the patient for the publication of photographs and medical data.

## Case presentation

A 27-year-old male presented to the Outpatient Department with the chief complaint of missing teeth in the upper anterior region since childhood. The patient reported occasional pus discharge from the maxillary anterior tooth region for the past five months. His medical history was significant for hydrocephalus diagnosed in childhood, which had been managed conservatively without surgical intervention. There was no reported family history of similar skeletal or dental anomalies, and the patient denied any history of trauma replacement, trauma, or systemic illnesses other than hydrocephalus.

**Figure 1:**
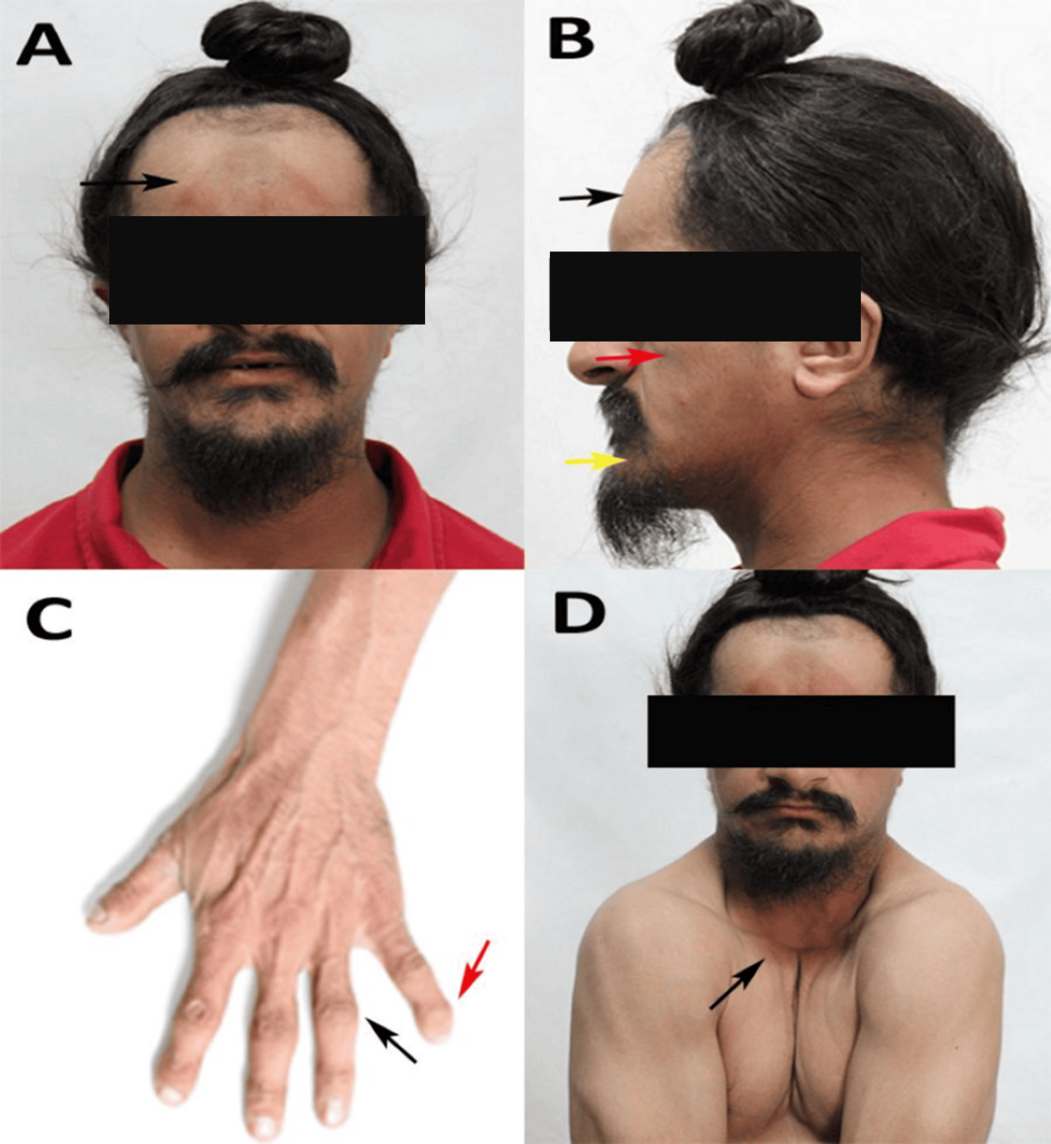
Clinical examination of the patient (A) Front view showing broad forehead (black arrow) indicative of frontal and parietal bossing. (B) Lateral view demonstrating frontal bossing (black arrow), midfacial hypoplasia (red arrow), and mandibular prognathism with a prominent chin (yellow arrow). (C) Hand examination revealing clinodactyly (red arrow) and hypoplastic phalanges (black arrow). (D) Demonstration of shoulder hypermobility with medial approximation due to clavicular hypoplasia (black arrow)

On general examination, the patient exhibited short stature (height approximately 150 cm), moderate build, and was well-nourished. He was well-coordinated, with no apparent neurological deficits. Extraoral examination revealed characteristic craniofacial features consistent with CCD. These included brachycephaly, frontal, occipital, and parietal bossing, with a broad forehead (Figure 1A). Mid-facial hypoplasia, mandibular prognathism, a prominent chin, and a concave facial profile with competent lips were noted (Figure 1B). Examination of the hands revealed hypoplastic and widened phalanges, clinodactyly of the fifth finger (Figure 1C), hypoplastic terminal phalanges, and shortened middle phalanges. The mental status was normal, with an age-appropriate IQ. The patient’s shoulders were narrow and markedly drooping, with hypermobility approximating both shoulders to the midline of the chest (Figure 1D).

**Figure 2:**
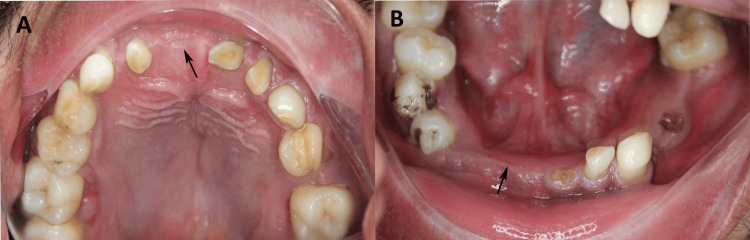
Intraoral examination (A) Maxillary arch showing multiple missing teeth. (B) Mandibular arch with multiple missing teeth

Intraoral examination revealed multiple missing teeth in both the maxillary and mandibular arches, along with retained deciduous teeth (Figure 2).

**Figure 3:**
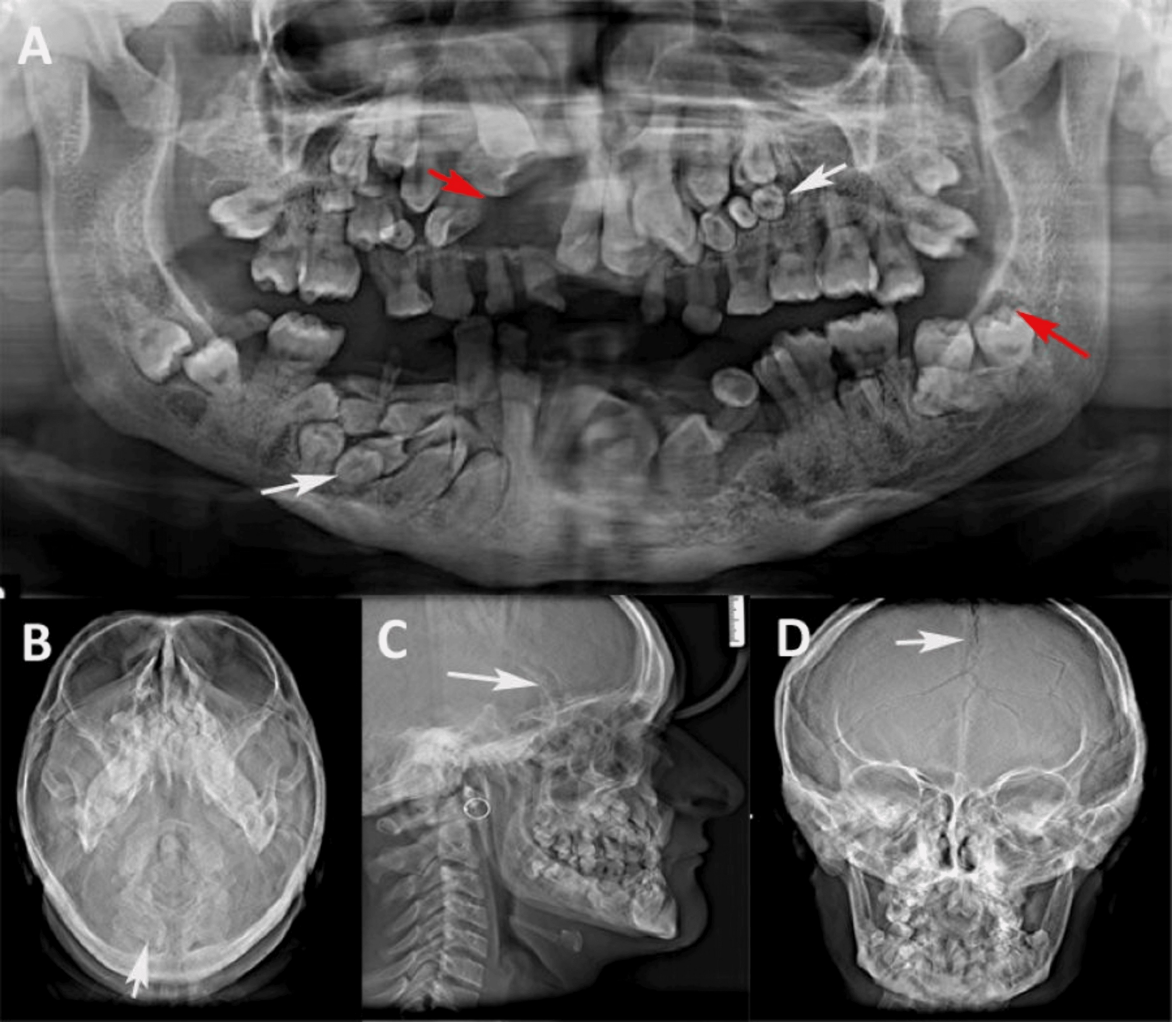
Radiographic features (A) Orthopantomogram (OPG) revealing multiple retained deciduous, impacted permanent, and supernumerary teeth (white arrows), with radiolucency (red arrows) indicating cystic changes. (B) Paranasal sinus (PNS) view showing underdeveloped sinuses and increased sutural markings on the skull. (C) Lateral cephalogram displaying a hypoplastic maxilla, prognathic mandible, numerous impacted teeth, and delayed cranial suture closure (white arrow). (D) Posteroanterior skull radiograph showing open cranial sutures and fontanelles (white arrow)

Anterior and posterior crossbites were also observed. Mild swelling was observed in the gingiva of the labial sulcus, causing vestibular obliteration in the maxillary right anterior region, extending from the midline to the right premolar region. The tongue and palate appeared normal, with no signs of clefting or a high-arched palate. Radiographic investigations were conducted to evaluate the extent of dental and skeletal abnormalities. OPG revealed retained deciduous teeth and multiple impacted permanent and supernumerary teeth in the maxillary and mandibular anterior, right, and left posterior regions (Figure 3A). A uniform, well-defined radiolucency in the maxillary right anterior region, displacing the impacted teeth toward the maxillary antrum, suggested a dentigerous cyst. Enlarged follicular spaces around the impacted teeth in the maxillary left region, mandibular right and left parasymphysis, and body of the mandible indicated a possible cystic transformation. The root apices of the mandibular supernumerary teeth were bilaterally located near the inferior border of the mandible in the parasymphysis and right posterior body regions. The mandible exhibited parallel-sided ascending rami, upward-pointing coronoid processes, and a rounded contour at the angle. 

**Figure 4:**
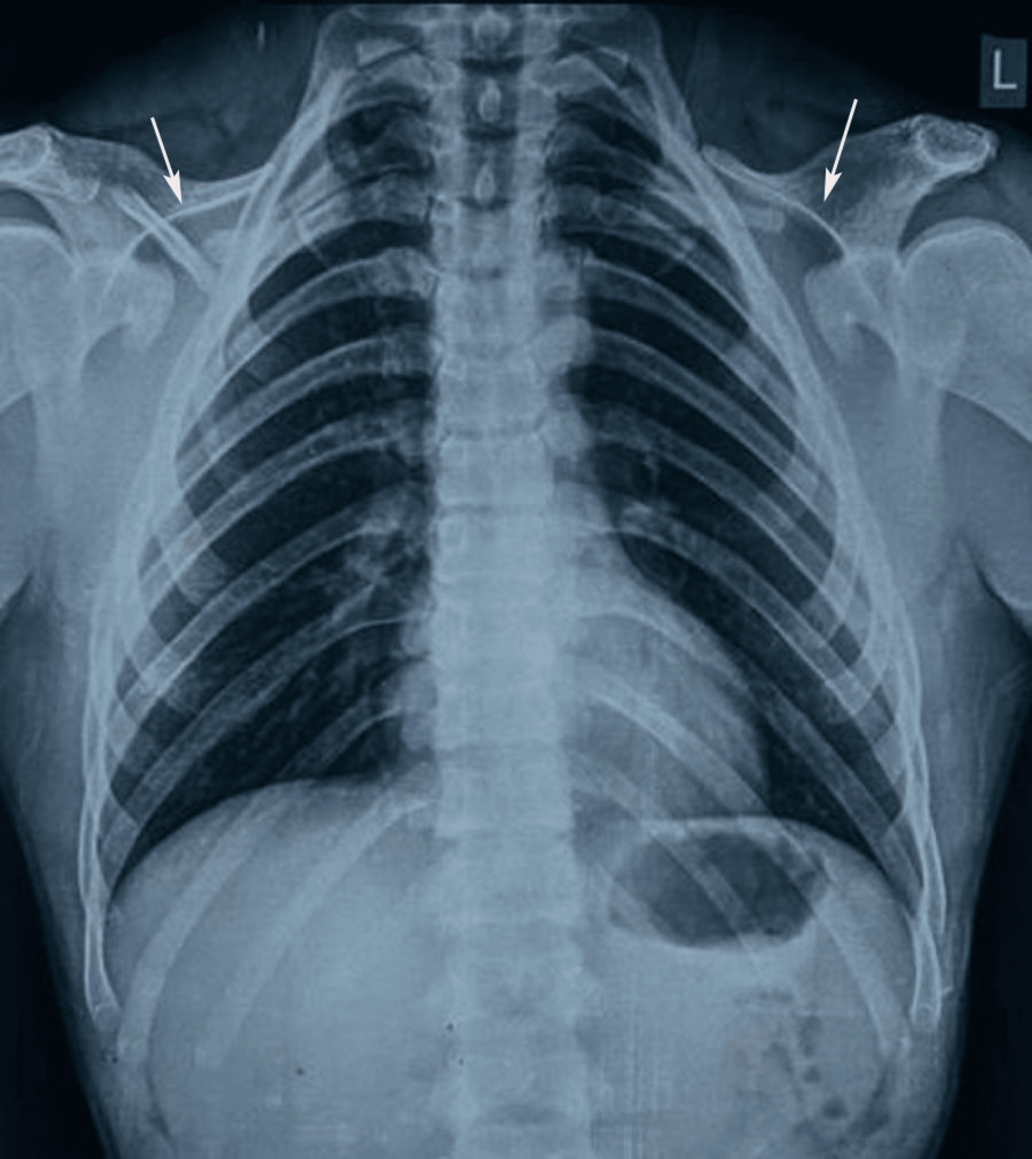
Chest X-ray showing bilateral hypoplastic clavicles (white arrows), a characteristic feature of cleidocranial dysplasia

Additional radiographic evaluations were performed to assess the extent of skeletal anomalies that are commonly associated with CCD. A paranasal sinus (PNS) view revealed poorly developed frontal and maxillary sinuses, consistent with hypoplastic paranasal structures typical of CCD (Figure 3B). The lateral cephalogram revealed a hypoplastic maxilla, increased cranial base angle, and multiple impacted teeth in both jaws, contributing to the patient's concave facial profile and mandibular prognathism (Figure 3C). Furthermore, posteroanterior (PA) skull radiography revealed open cranial sutures and fontanelles, indicating delayed ossification (Figure 3D) and incomplete fusion of the calvarial bones, which are hallmark features of CCD. Chest X-ray revealed bilateral hypoplastic clavicles, a characteristic feature of CCD. The clavicles appeared shortened and underdeveloped, with poor visualization of the medial ends and a lack of articulation with the sternum, contributing to shoulder hypermobility and the ability to approximate the shoulders toward the midline (Figure 4).

**Figure 5:**
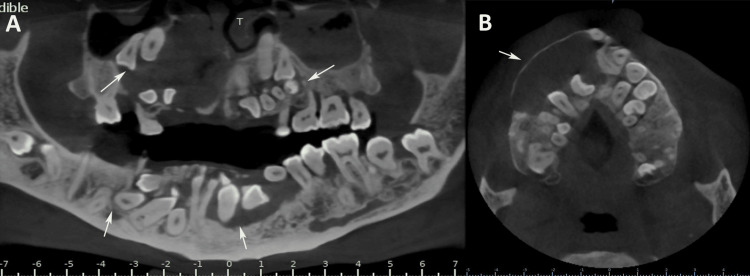
CBCT findings (A) Panoramic CBCT reconstruction revealing approximately 45 impacted teeth with cystic changes in the mandible and proximity of root apices to the inferior mandibular border. (B) Axial CBCT sections showing a large dentigerous cyst in the right maxilla involving teeth from the right upper central incisor to the first molar, with cortical plate breach and nasal cavity involvement CBCT: cone beam computed tomography

CBCT confirmed approximately 45 impacted teeth in various positions (vertical, mesio-angular, horizontal, and disto-angular), including multiple supernumerary teeth with cystic degeneration around several teeth. A well-defined hypodense lesion in the right maxilla (involving all right upper teeth from the central incisor to the first molar) measured 23.29 mm (superoinferior), 30.28 mm (mesiodistal), and 28.98 mm (buccopalatal), suggestive of a dentigerous cyst. The lesion had smooth, distinct margins and a completely hypodense internal structure. Superior displacement of the impacted teeth toward the nasal cavity with perforation of the nasal cavity floor was observed. Expansion and discontinuity of the buccal and palatal cortical plates were observed in the right maxilla. Additional cystic transformations were evident in the right and left mandibles, with a breach of the buccal cortical plate in relation to the left lower canine, first and second premolars, and first molar teeth. Multiple impacted teeth were located bilaterally below the mandibular canal, with the root apices approximating the inferior border of the mandible (Figure 5).

Based solely on clinical and radiological findings, the differential diagnoses included pycnodysostosis (dense bones, short stature, but typically no supernumerary teeth or cysts), Crane-Heise syndrome (clavicular aplasia and craniofacial defects, but usually fatal in infancy), Yunis-Varon syndrome (clavicular hypoplasia and digital anomalies, but with severe microcephaly and developmental delay), and Craniosynostosis-Anal anomalies-Porokeratosis syndrome (CDAGS) syndrome (craniosynostosis and porokeratosis, but lacking extensive dental anomalies). Extensive supernumerary teeth and dentigerous cysts strongly favor the diagnosis of CCD.

Specific diagnostic tests are recommended to confirm the etiology and diagnosis of CCD, specific diagnostic tests were recommended. Genetic testing was performed to identify mutations in the *RUNX2* gene on chromosome 6p21 using the procedure described by Çamtosun et al. [8], which is critical for osteoblast differentiation and bone formation. Whole-exome sequencing or targeted gene panel analysis of a peripheral blood sample revealed a heterozygous missense mutation in the *RUNX2* gene, confirming the molecular diagnosis of CCD. Additionally, a complete blood count (CBC) and serum biochemical profile, including alkaline phosphatase (ALP), calcium, and vitamin D levels, were assessed to evaluate bone metabolism and rule out other metabolic bone disorders such as hypophosphatasia. The CBC was within normal limits, serum ALP was mildly elevated, consistent with active bone remodeling, and calcium and vitamin D levels were normal, excluding hypophosphatasia or vitamin D deficiency. These findings support the diagnosis of CCD rather than differential diagnoses such as pycnodysostosis, which may present with overlapping skeletal features but lack specific dental and *RUNX2 *gene-related findings.

Based on the clinical, radiographic, and genetic findings, a definitive diagnosis of CCD was established. The patient was referred to the Department of Oral Surgery for surgical management of the dentigerous cysts and impacted teeth, with a coordinated treatment plan involving the Departments of Orthodontics and Prosthodontics to address extensive dental anomalies and skeletal deformities.

## Discussion

CCD manifests as a spectrum of skeletal and dental anomalies, with dental features often including supernumerary teeth, delayed eruption, and dentigerous cysts. Our case involved a 27-year-old male with 45 impacted teeth, multiple dentigerous cysts, and classic skeletal features such as hypoplastic clavicles and open cranial sutures, representing an exceptionally severe presentation compared to previously reported cases. The extent of dental involvement and cystic transformation distinguishes this case as rare and complex.

The literature on CCD typically reports 6-20 supernumerary teeth, with the impaction of permanent teeth attributed to mechanical obstruction, lack of cellular cementum, or fibrous barriers in the dental follicle. For example, Granado-Abasto et al. [9] documented two cases with multiple supernumerary teeth and impacted permanent teeth; however, further details were not reported because of the absence of proper radiological records. No dentigerous cysts were detected. Similarly, González López et al. [10] reported a familial case with 8-10 supernumerary teeth per individual (one mother and two adolescent girls), with no mention of cystic changes as extensive as those in our case. The presence of 45 impacted teeth, including both permanent and supernumerary teeth, is rare, as most cases do not exceed 30 teeth.

The patient was of short stature and exhibited skeletal underdevelopment. This finding was supported by Jensen [11], who conducted a study on the somatic growth of 17 Danish patients with CCD, whose ages ranged from 5 to 46 years. The heights of six males and eight females were recorded and subsequently compared with the established Danish reference standards. These findings indicate a notable incidence of growth retardation, particularly in females. The heights of males with CCD were predominantly between the 5th and 50th percentiles, whereas all females with CCD exhibited heights below the 5th percentile. Yoshida et al. [12] investigated genotype-phenotype correlations among 17 Japanese patients diagnosed with CCD. Their findings indicated that mutations in the *RUNX2* gene affecting the Runt domain, which plays a crucial role in DNA binding, are associated with both short stature and disease severity. Furthermore, they demonstrated that patients exhibited normal height when harboring mutations in which the Runt domain remained unchanged. The mutation in the *RUNX2* gene identified in our patient constituted a missense mutation, resulting in a modification of the 155th amino acid in exon 4, situated within the Runt domain, leading to a short stature.

The presence of 45 impacted teeth and multiple dentigerous cysts in our patient likely stemmed from a severe disruption in *RUNX2* gene function, which regulates odontogenesis and bone development [3]. *RUNX2* gene mutation, confirmed via genetic analysis, impairs the differentiation of the dental follicle mesenchyme and periodontal ligament, leading to hyperplasia of dental lamina remnants and the formation of excessive supernumerary teeth [2,3]. These teeth, often in anomalous positions, cause mechanical obstruction, prevent eruption, and promote cystic degeneration of the follicular sac, resulting in dentigerous cysts [13]. The large cyst in the right maxilla, along with additional cystic transformations in the mandible, may be exacerbated by prolonged impaction and altered bone resorption dynamics, which are characteristic of CCD and further predispose the patient to cystic changes around the impacted teeth.

The multiple dentigerous cysts in our patient, particularly the large cyst in the right maxilla (23.29 mm × 30.28 mm × 28.98 mm) with cortical plate breaches and nasal cavity involvement, further differentiate this case. Pereira et al. [13] documented two instances of dentigerous cysts located in the mandible; however, the specific dimensions were not provided, and detection was performed solely using OPGs. Hemalatha and Balasubramaniam [14] described a case of CCD with a single dentigerous cyst associated with an impacted tooth; however, multiple cysts with extensive bone involvement have seldom been reported. The cystic transformation in our patient, affecting both the maxillary and mandibular regions, suggests a pronounced disturbance in tooth eruption pathways, possibly linked to severe *RUNX2* gene mutation effects on the dental follicle mesenchyme [3].

The genetic confirmation of our case via *RUNX2* gene mutation analysis aligns with that of Mundlos [2], who emphasized the role of the *RUNX2* gene in osteoblast differentiation and tooth eruption. However, the severity of the dental anomalies suggests a potentially novel mutation or modifier gene, warranting further genetic research. Unlike Roberts et al. [15], who described CCD cases with primary orthodontic challenges, our patient’s presentation necessitates a broader multidisciplinary approach due to extensive cystic lesions and skeletal deformities.

Extensive dental and cystic involvement underscores the need for early diagnosis through radiographic and genetic testing to prevent complications such as cyst expansion or bone resorption. Multidisciplinary management involving oral surgery, orthodontics, and prosthodontics is critical for functional and aesthetic restoration [16]. Limitations of this report included the lack of family history analysis to confirm inheritance patterns and the absence of long-term follow-up data to assess treatment outcomes, which could inform future management strategies.

## Conclusions

This report highlights a rare and severe presentation of CCD in a 27-year-old male with 45 impacted supernumerary and permanent teeth, multiple dentigerous cysts, and characteristic skeletal anomalies, including hypoplastic clavicles, open cranial sutures, and short stature. Extensive dental involvement, confirmed by OPG and CBCT, and molecular diagnosis via *RUNX2* gene mutation analysis underscore the complexity of this case. The presence of large cystic lesions with cortical breaches emphasizes the critical need for early diagnosis to prevent complications such as bone resorption or infection. Multidisciplinary management involving oral surgery, orthodontics, and prosthodontics is essential to address the functional and aesthetic challenges posed by dental and skeletal abnormalities. This report reinforces the importance of comprehensive clinical, radiographic, and genetic evaluations in the management of CCD to optimize patient outcomes.
